# Chemoenzymatic Synthesis of *trans*-β-Aryl-δ-hydroxy-γ-lactones and Enzymatic Kinetic Resolution of Their Racemic Mixtures

**DOI:** 10.3390/molecules21111552

**Published:** 2016-11-23

**Authors:** Andrzej Skrobiszewski, Witold Gładkowski, Gabriela Maciejewska, Czesław Wawrzeńczyk

**Affiliations:** 1 Department of Chemistry, Wrocław University of Environmental and Life Sciences, Norwida 25, Wrocław 50-375, Poland; glado@poczta.fm (W.G.); czeslaw.wawrzenczyk@up.wroc.pl (C.W.); 2 Central Laboratory of the Instrumental Analysis, Wrocław University of Technology, Wybrzeże Wyspiańskiego 27, Wrocław 50-370, Poland; gabriela.maciejewska@pwr.edu.pl

**Keywords:** hydroxylactones, kinetic resolution, lipases

## Abstract

Two novel and convenient routes to obtain enantiomerically enriched *trans*-β-aryl-δ-hydroxy-γ-lactones **5a**–**d** with potential antifeedant and anticancer activity were developed. In the first method starting from corresponding enantiomers of γ,δ-unsaturated esters **4a**–**d** derived from enzymatically resolved allyl alcohols **1a**–**d**, both enantiomers of hydroxylactones **5a**–**d** were synthesized with high enantiomeric excesses (73%–97%). Configurations of the stereogenic centers of the synthesized compounds were assigned based on the mechanism of acidic lactonization of esters **4a**–**d** in the presence of *m*-chloroperbenzoic acid (*m*-CPBA). An alternative method for the production of optically active *trans-*β-aryl-δ-hydroxy-γ-lactones **5a**–**d** was lipase-catalyzed kinetic resolution of their racemic mixtures by transesterification with vinyl propionate as the acyl donor. The most efficient enzyme in the screening procedure was lipase B from *Candida antarctica*. Its application on a preparative scale after 6 h afforded unreacted (+)-(4*S*,5*R*,6*S*)-hydroxylactones **5a**–**d** and (+)-(4*R*,5*S*,6*R*)-propionates **6a**–**d**, most of them with high enantiomeric excesses (92%–98%). Resolution of lactone **5d** with bulky 1,3-benzodioxol ring provided products with significantly lower optical purity (*ee* = 89% and 84% for hydroxylactone **5d** and propionate **6d**, respectively). The elaborated methods give access to both enantiomers of *trans-*β-aryl-δ-hydroxy-γ-lactones **5a**–**d** with the defined absolute configurations of stereogenic centers, which is crucial requirement for the investigations of relationship: spatial structure–biological activity.

## 1. Introduction

Lactones with an aromatic substituent are widely present in the plant kingdom. They are isolated from roots, tubers, knots, stalks, seeds and fruits [1,2,3] and have also been found in mushrooms and cyanobacteria [4,5]. They are reported to display a wide variety of biological activities such as anticancer [6,7], antioxidant [8,9], antifungal [10,11], antiviral [12,13], anti-inflammatory [14], antifeedant [15], anticonvulsant [16] and phytoestrogenic [17,18].

Optically active lactones are of high importance due to their application as building blocks in natural therapeutic compounds [19,20] and the well-known relationship between the configuration of stereogenic centers and their biological activity [21,22]. Therefore, there is a growing need to develop new methods for the synthesis of enantiomerically enriched lactones. These strategies involve *i.a.* alkylation of chiral precursors [23,24], catalytic asymmetric hydrogenation of butenolides [25], kinetic resolution of lactone precursors by hydrolysis of ester bond [26,27] and different whole-cell mediated reactions including reduction of unsaturated formyl esters [28], stereoselective reduction of a carbonyl group in γ-acetyl-γ-lactones [29] and enantioselective hydrolysis of nitriles [30].

With regard to these reports, we have taken up research on the synthesis of a series of racemic γ-lactones with various β-phenyl substituents at the lactone ring [31,32,33,34,35]. We have also developed convenient chemoenzymatic methods for the synthesis of optically active iodolactones with antiproliferative activity starting from enantiomerically enriched allyl alcohols with 4-arylbut-3-en-2-ol system as chiral precursors [36,37,38]. In this paper, we would like to present two novel, alternative methods for the production of optically active *trans*-β-aryl-δ-hydroxy-γ-lactones. One of these methods involves the application of chiral precursors, and an alternative method is based on lipase-catalyzed kinetic resolution of racemic mixtures in the transesterification process.

## 2. Results and Discussion

In our previous paper [33] using methodology reported earlier by Gowri-Shankar [39], we reported the synthesis of racemic *trans*-δ-hydroxy-γ-lactones **5a**–**d** as the main products of lactonization of corresponding of γ,δ-unsaturated esters **4a**–**d**. Because of their proven biological activities, i.e., antifeedant and antiproliferative, we decided to evaluate methods for the production of these compounds in an optically active form.

**Scheme 1:**
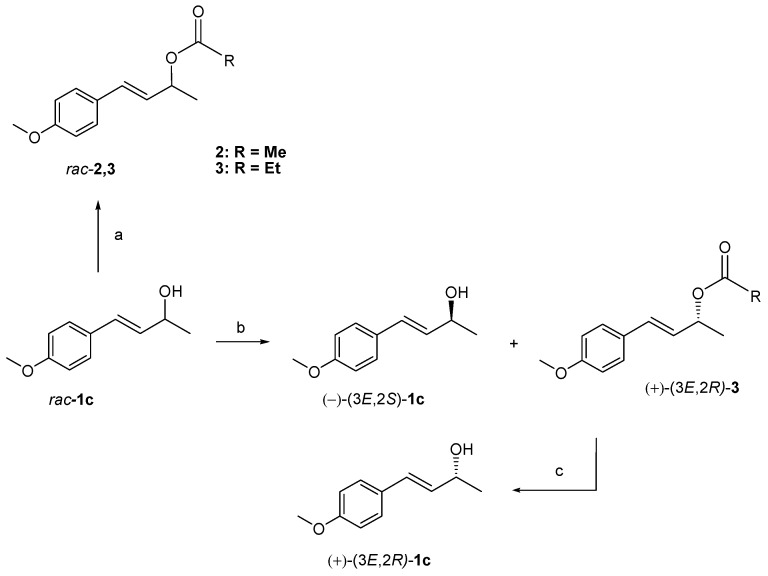
Enzymatic resolution of racemic 4-(4’-methoxyphenyl)but-3-en-2-ol and synthesis of racemic esters **2**,**3**. *Reagents and conditions*: (a) MeC(O)Cl or EtC(O)Cl, Et_2_O, pyridine, 0 °C to r.t., 24 h; (b) CAL-B, vinyl propionate, DIPE, r.t., 4 h; and (c) 5% NaOH, reflux, 3 h.

The first elaborated method was based on the lactonization of chiral precursors, enantiomerically enriched γ,δ-unsaturated esters **4a**–**d** in the presence of *m*-CPBA. These precursors are products of the stereoselective Claisen–Johnson rearrangement of enzymatically resolved allyl alcohols **1a**–**d**. During this reaction, a complete transfer of chirality from atom C-2 of alcohols to the benzylic position C-3 of ester is observed. The details of this two-step synthesis involving lipase-catalyzed enzymatic resolution of allyl alcohols **4a**,**b**,**d** and their orthoester Claisen rearrangement were reported earlier for synthesis of enantiomeric pairs of esters with unsubstituted phenyl ring (**4a**), *p*-methylphenyl substituent (**4b**) and 1,3-benzodioxol ring (**4d**) [36,38]. In order to obtain both enantiomers of ester **4c** with *p*-methoxyphenyl substituent, a kinetic resolution of racemic alcohol **1c** via transesterification process catalyzed by lipase B from *C. antarctica* (CAL-B) was applied (Scheme 1). Reaction was monitored by chiral GC after derivatization of unreacted alcohol into acetate. Standards used in this analysis—racemic acetate **2** and propionate **3**—were synthesized by the esterification of *rac*-**1c** with acetyl and propionyl chloride, respectively (Section 3.3).

**Scheme 2:**
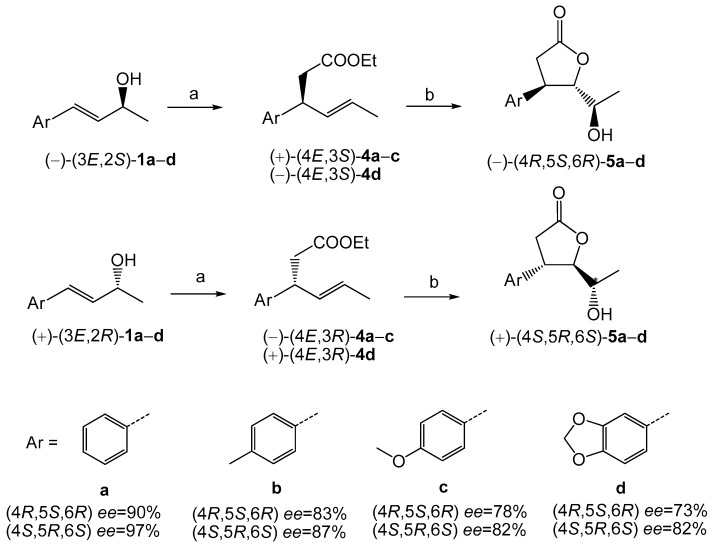
Synthesis of optically active hydroxylactones **5a**−**d** from enantiomerically enriched alcohols **1a**−**d**. *Reagents and conditions*: (a) CH_3_C(OEt)_3_, propionic acid, 138 °C, 24 h; and (b) *m*-CPBA, CF_3_COOH, CHCl_3_, 24 h.

Vinyl propionate was used as the acyl donor because it was successfully used in the resolution of analogs of **1c** with 4-phenylbut-3-en-2-ol system [36,37,38]. After 4 h (−)-(3*E*,2*S*)-4-(4′-methoxyphenyl)but-3-en-2-ol (**1c**) and its propionate (+)-(3*E*,2*R*)-**3** were obtained in 45% and 43% yield and 78% and 82% *ee*, respectively. Products were separated by column chromatography. The configuration *S* of slowly reacting enantiomer of alcohol **1c** was confirmed by the negative specific rotation sign corresponding with that reported in the literature for the known (*S*)-alcohol [40]. Consequently, the configuration of propionate (+)-**3**, product of faster reacting enantiomer of alcohol **1c**, was assigned as *R*. Propionate (+)-(3*E*,2*R*)-**3**, which was not previously obtained in optically active form, was subsequently hydrolyzed under basic conditions to (+)-(3*E*,2*R*)-alcohol **1c** (yield 98%, *ee* = 82%). Enantiomerically enriched alcohols (−)-(3*E*,2*S*)-**1c** and (+)-(3*E*,2*R*)-**1c** were subjected to Johnson–Claisen rearrangement. As expected, the reactions proceeded with complete retention of configuration of both stereogenic center and double bond. In the result, respective enantiomers of ester **4c**: (+)-(4*E*,3*S*) and (−)-(4*E*,3*R*) with exactly the same enantiomeric excesses as starting alcohols were obtained as the only products (Scheme 2).

Both enantiomeric forms of four γ,δ-unsaturated esters **4a**–**d** were subjected to lactonization with *m*-CPBA and catalytic amount of trifluoroacetic acid (Scheme 2).

**Scheme 3:**
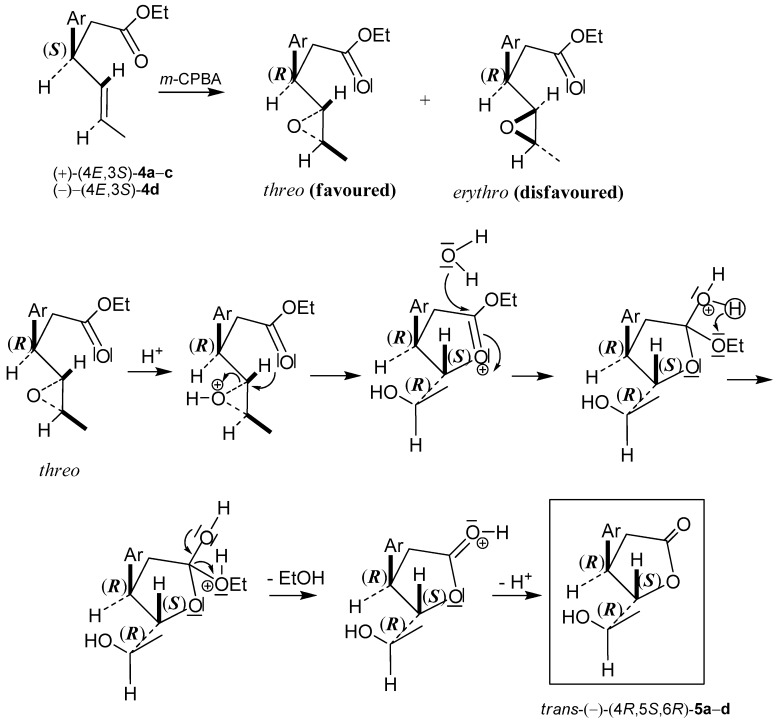
Formation of two epoxyesters from esters (4*E*,3*S*)-**4a**–**d** and mechanism of acidic lactonization of favoured *threo* isomer showing the configurations of stereogenic centers of isolated *trans*-δ-hydroxy-γ-lactones **5a**–**d**.

During the reaction two epoxyesters, *threo* and *erythro* are formed and four possible lactones could be the final products: two γ-lactones and two δ-lactones (Figure S1). Unfortunately, similarly to the reaction carried out earlier for racemic compounds [33], δ-lactones were isolated as the unseparable mixture of stereoisomers. *cis* γ-Lactones formed from *erythro* epoxyesters were not isolated and the only isomers isolated in pure form were *trans* γ-lactones **5a**–**d,** which are the products of lactonization of *threo* epoxyesters. Advantageous formation of *trans* isomers **5a**–**d** over the *cis* isomers can be explained by the fact that in *threo* epoxyesters oxygen of the epoxide ring is less hindered by large aromatic substituent compared with *erythro* epoxyesters (Scheme 3).

Chiral GC analysis indicated that enantiomeric excesses of lactones **5a**–**d** corresponded with those determined earlier for starting esters **4a**–**d** and ranged from 73% to 90% in the case of hydroxylactones obtained from (*S*)-esters **4a**–**d** and 82%–97% for their antipodes synthesized from (*R*)-esters **4a**–**d**. The important challenge was the assignment of the configurations of stereogenic centers at C-4, C-5 and C-6 for newly synthesized enantiomers, which was made based on the mechanism of acidic lactonization (Scheme 3). Monitoring the composition of the reaction mixture did not show the presence of possible intermediate diolester, which excluded the opening of epoxide ring by water and confirmed the mechanism of lactonization proposed by Olejniczak et al. [41]. This mechanism involves the protonation of an oxirane ring followed by its nucleophilic opening by the attack of an oxygen from carboethoxy group with the simultaneous formation of hydroxy group. In the next step of reaction, nucleophilic addition of water to carboethoxy group takes place with subsequent release of the ethanol. Considering lactonization of (*S*)-esters **4a**–**d** (Scheme 3), the configuration *R* at C-4 of forming *trans*-(−)-δ-hydroxy-γ-lactones **5a**–**d** is the result of the configuration of starting ester, and its apparent change is only the result of different priority of substituents after formation of oxirane ring. The configurations *S* at C-5 and *R* at C-6 are the consequence of stereochemical course of reaction in which the carboethoxy group approaches the C-5 atom from the opposite side of the oxirane ring. Thus, the C-O bond of the γ-lactone ring and hydroxy group are oriented antiperiplanary. Similar reasoning let us assign the configuration of (+)-δ-hydroxy-γ-lactones **5a**–**d** formed from (*R*)-esters **4a**–**d** as 4*S*,5*R* and 6*S*. The presented stereochemical course of reaction was earlier confirmed for the products of lactonization of ethyl esters of 3,7-dimethyl-4,5-epoxyoctanoic acid and its 7-methyl homolog, in which the configurations of stereocenters were determined by CD (Circular Dichroism) measurements [42].

**Scheme 4:**
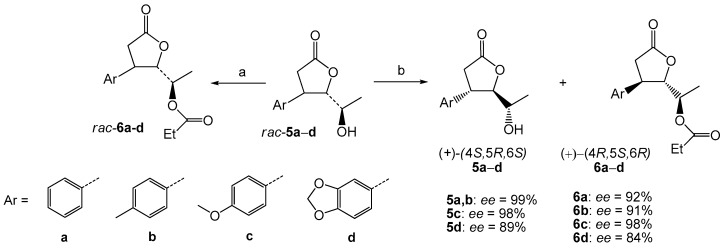
Enzymatic resolution of racemic hydroxylactones **5a**−**d**. *Reagents and conditions*: (a) EtC(O)Cl, Et_2_O, pyridine, 0 °C to r.t., 24 h; and (b) CAL-B, vinyl propionate, DIPE, r.t., 2 h.

The alternative method for the production of *trans*-β-aryl-δ-hydroxy-γ-lactones **5a**–**d** in enantiomerically enriched form was kinetic resolution of their racemic forms synthesized previously [33]. Due to the large difference in sizes of two substituents at the stereogenic center C-6 joined with a secondary hydroxy group, the hydroxylactones **5a**–**d** seemed to be excellent substrates for the lipase-catalyzed transesterification [43,44], and high enantiomeric excesses of the products were expected. In the screening procedure, racemic *trans*-β-phenyl-δ-hydroxy-γ-lactone **5a** as a model substrate was subjected to the action of four commercially available lipases in the presence of vinyl propionate as the acyl donor (Scheme 4). The reaction was conducted in diisopropyl ether (DIPE) and monitored by chiral gas chromatography with racemic lactones **5a**–**d** [33] and racemic propionates **6a**–**d** (Section 3.3) as the standards. The results are shown in Table 1.

The most effective biocatalyst was CAL-B. At 49% conversion after 1 h of the reaction catalyzed by this enzyme, the enantiomeric excesses of the slower reacting (+)-enantiomer of δ-hydroxy-γ-lactone **5a** and its propionate (+)-**6a** were 96% and 99% respectively. Continuing the process, a slight decrease of *ee* for (+)-propionate **6a** was observed, reaching 96% after 6 h, whereas *ee* of hydroxylactone **5a** raised to 99%. Good results were achieved for Lipozyme TL IM (*Thermomyces lanuginosus* lipase) as well, but the transesterification proceeded at a lower rate. After 6 h, the 53% conversion was observed. During this period, the enantiomeric excess of unreacted (+)-hydroxylactone **5a** gradually increased from 12% to 91%, whereas the optical purity of (+)-propionate **6a** decreased from 99% to 80%. High enantioselectivity (E > 200) was found for Amano PS (lipase from *Burkholderia cepacia*). In this case, high enantiomeric enrichment of (+)-propionate **6a** was found (*ee* = 99%), but the *ee* of unreacted (+)-hydroxylactone **5a** even after 6 h was unsatisfactory (65%). After 6 h of the process catalyzed by CCL low (*ee* = 55%) and moderate (*ee* = 71%), enantiomeric purity of substrate **5a** and ester **6a**, respectively, was observed at the 44% conversion. For all enzymes, prolonging reaction time affected neither conversion nor enantiomeric composition of the products.

Taking into consideration the highest enantiomeric excesses obtained after 6 h for both hydroxylactone **5a** and propionate **6a**, CAL-B was selected as the biocatalyst for the kinetic resolution of hydroxylactones **5a**–**d** on a preparative scale (Table 2). After 6 h of reaction, the conversion of the substrates in all cases was 50%–52%. The unreacted substrates **5a**–**d** and their propionates **6a**–**d** were separated by column chromatography in yields ranging from 34% to 45%, respectively, which is a very satisfactory result for kinetic resolution. (+)-δ-Hydroxy-γ-lactone **5a** with unsubstituted phenyl ring and its (+)-propionate **6a** were obtained with a very high enantiomeric excesses, 99% and 92%, respectively. Taking into consideration the increasing size of an aryl substituent at β-position in substrates **5b**–**d**, comparably high enantioselectivity of the kinetic resolution was expected. It was confirmed in the case of (+)-hydroxylactones with *p*-methylphenyl- and *p*-methoxyphenyl ring (**5b** and **5c**) (*ee* = 99% and 98%, respectively) and their corresponding (+)-propionates **6b** and **6c** (*ee* = 91% and 98%, respectively). The presence of additional 1,3-benzodioxol ring condensed with benzene resulted in the lowest effectiveness of kinetic resolution for hydroxylactone **5d**, of which (+)-enantiomer was obtained with *ee* = 89%. Optical purity of its (+)-propionate **6d** was comparably low (*ee* = 84%). A similar effect of the 1,3-benzodioxol ring on the enantioselectivity of lipase-catalyzed transesterification with vinyl propionate was also reported for allyl alcohol **1d** [37]. It is likely that the steric hindrance in the molecules alcohol **1d** and hydroxylactone **5d** impedes the binding of these substrates to the active site of the lipase.

Comparison of the specific rotation signs of slowly transesterified δ-hydroxy-γ-lactones **5a**–**d** and those found for isomers with defined absolute configurations synthesized earlier from (*R*)- and (*S*)-esters **4a**–**d** let us establish undoubtedly the absolute configurations of both products of enzymatic transesterification. Slowly reacting (+)-δ-hydroxy-γ-lactones **5a**–**d** possessed configurations 4*S*,5*R*,6*S*, and opposite configurations 4*R*,5*S*,6*R* were consequently ascribed to (+)-propionates **6a**–**d**. The configuration of these products at C-6 is consistent with that predicted based on the Kazlauskas’ rule. In this case, taking into consideration that the order of substituents at stereogenic center C-6 follows the rule: OH > large substituent (β-aryl substituted γ-lactone ring) > medium substituent (methyl group), lipases preferentially catalyze the esterification of isomers with configuration 6*R*, leaving those with 6*S* configuration untouched.

## 3. Experimental Section

### 3.1. General

Analytical thin layer chromatography (TLC) was performed on silica gel-coated aluminum plates (DC-Alufolien Kieselgel 60 F_254_, Merck, Darmstadt, Germany), and the chromatograms were developed using hexane/acetone mixture (4:1, *v*/*v*) as the developing system. Compounds were detected by submersing the plates in a solution of 2 g H_3_[P(Mo_3_O_10_)_4_] and 1 g Ce(SO_4_)_2_ in 100 mL 10% H_2_SO_4_.

The course of reactions was monitored by gas chromatography (GC) on Agilent Technologies (Palo Alto, CA, USA) 6890N with hydrogen as the gas carrier and autosampler instrument using DB-5HT column (polyimide-coated fused silica tubing, 30 m × 0.25 mm × 0.10 µm). The temperature programme was as follows: injector 220 °C, detector (FID) 330 °C, initial column temperature column 90 °C, 90–330 °C (rate 20 °C·min^−1^), final column temperature 330 °C (hold 2 min).

Enantiomeric compositions of synthesized compounds were determined by chiral gas chromatography (CGC, Agilent Technologies) using CP (Chrompack)-Chiral-Dex CB (Cyclodextrin Beta) column (25 m × 0.25 mm × 0.25 μm, Varian, Palo Alto, CA, USA). The temperature programme was as follows: injector 260 °C, detector (FID) 280 °C; for acetate **2** and propionate **2**: initial column temperature 80 °C, 80–200 °C (5 °C·min^−1^), final column temperature 200 °C (1 min); for esters **4a**–**d**: initial column temperature 50 °C, 80–200 °C (0.5 °C·min^−1^), final column temperature 200 °C (1 min); for hydroxylactones **5a**–**d** and the propionates **6a**–**d**: initial column temperature: 80 °C, 80–200 °C (3 °C·min^−1^), final column temperature 200 °C (1 min). Racemic acetate **2** and racemic propionates **3** and **6a**–**d** were used as standards.

Products of synthesis and enzymatic reactions were separated by preparative column chromatography on silica gel (Kieselgel 60, 230–400 mesh, Merck) using mixtures of various organic solvents as a mobile phase.

The NMR spectra (^1^H-NMR, ^13^C-NMR, HMBC and HMQC) were recorded in a CDCl_3_ solution on a Bruker Avance II 600 MHz spectrometer (Bruker, Rheinstetten, Germany). The IR spectra were determined using a Mattson IR 300 Thermo Nicolet spectrophotometer (Mattson, Waltham, MA, USA) using KBr pellets or as neats. High resolution mass spectra (HRMS) were recorded using electron spray ionization (ESI) technique on spectrometer Waters ESI-QTOF Premier XE (Waters Corp., Millford, MA, USA). The optical rotations were measured on a Jasco P-2000-Na digital polarimeter (Easton, PA, USA) with an intelligent Remote Module (iRM) controller. The indexes of refraction were measured on an Abbe refractometer (Carl Zeiss, Jena, Germany). Melting points were determined on Boetius apparatus (Nagema, Germany).

### 3.2. Chemicals and Enzymes

Diisopropyl ether (purity ≥ 98.5%), propionyl chloride (purity 99%), acetyl chloride (purity ≥ 99%), vinyl propionate (purity 98%), triethyl orthoacetate (purity 97%), 3-chloroperbenzoic acid (purity ≤ 77%), trifluoroacetic acid (purity 99%) and diatomaceous earth (Celite 560) were purchased from Sigma-Aldrich (St. Louis, MO, USA). Analytical grade chemicals: propionic acid, sodium hydrogen carbonate, sodium hydroxide, anhydrous magnesium sulphate, sodium chloride and hydrochloric acid (35%–37%) and organic solvents of analytical grade were purchased from POCH (Gliwice, Poland). Racemic *trans*-β-aryl-δ-hydroxy-γ-lactones (**5a**–**d**) were synthesized from corresponding racemic γ,δ-unsaturated ethyl esters **4a**–**d** as described previously [33]. Racemic (*E*)-4-(4′-methoxyphenyl)but-3-en-2-ol (**1c**) was obtained from anisaldehyde according to the procedure described by Mazur et al. [31]. Enantiomerically enriched esters: (*S*)-**4a** (*ee* = 90%) and (*R*)-**4a** (*ee* = 97%), (*S*)-**4b** (*ee* = 83%) and (*R*)-**4b** (*ee* = 87%), (*S*)-**4d** (*ee* = 73%) and (*R*)-**4d** (*ee* = 82%) were obtained according to the procedures described by Gładkowski et al. [36,38]. Amano Lipase PS from *Burkholderia cepacia* (≥30,000 U/g), lipase B from *Candida antarctica* immobilized in a macroporous acrylic resin (CAL-B > 5000 U/g), lipase from *Candida cylindracea* (CCL, 5.18 U/mg) were purchased from Sigma-Aldrich and immobilized granulate *Thermomyces lanuginosus* lipase (Lipozyme TL IM, 250 U/g) from Novozymes (Bagsvaerd, Denmark).

### 3.3. General Procedure for the Synthesis of Racemic Esters ***2***, ***3*** and ***6a***–***d***

Racemic allyl alcohol **1c** and racemic β-aryl-δ-hydroxy-γ-lactones (**5a**–**d**) (0.003 mol) were esterified according to the standard procedure [37] using propionyl chloride or acetyl chloride in dry diethyl ether and dry pyridine (Scheme 1).

*(E)-4-(4′-Methoxyphenyl)but-3-en-2-yl acetate* **2**. Yield 83% (0.55 g); $n_{D}^{20}$ = 1.5439; spectroscopic data in accordance with those reported earlier [40].

*(E)-4-(4′-Methoxyphenyl)but-3-en-2-yl propionate* **3**. Yield 85% (0.6 g); $n_{D}^{20}$ = 1.5263; IR (film, cm^−1^): 1732 (s), 1608 (s), 1512 (s) 1249 (s), 1187 (s), 1036 (s), 968 (m), 808 (m); ^1^H-NMR (300 MHz, CDCl_3_) δ: 1.15 (t, *J* = 7.8 Hz, 3H, CH_3_CH_2_C(O)), 1.39 (d, *J* = 6.3 Hz, 3H, CH_3_-1), 2.34 (quartet, *J* = 7.8 Hz, 2H, CH_3_CH_2_C(O)), 3.80 (s, 3H, –OCH_3_), 5.52 (m, 1H, H-2), 6.05 (dd, *J* = 15.9, 6.9 Hz, 1H, H-3), 6.55 (d, *J* = 15.9 Hz, 1H, H-4), 6.84, 7.30 (two m, 4H, –C_6_H_4_); ^13^C-NMR (75 MHz, CDCl_3_) δ: 9.08 (CH_3_CH_2_C(O)), 20.42 (C-1), 27.88 (CH_3_CH_2_C(O)), 55.23 (–OCH_3_), 70.99 (C-2), 113.91, 127.73 (C-2′, C-3′, C-5′, C-6′), 126.66 (C-3), 129.05 (C-1′), 131.06 (C-4), 159.38 (C-4′), 173.76 (CH_3_CH_2_C(O)); HRMS (ESI): *m*/*z* calcd. for C_14_H_18_O_3_ [M + H]^+^: 235.1334, found 235.1339.

*4-Phenyl-5-(1-propionyloxyethyl)dihydrofuran-2-one* **6a**. Yield 87% (0.68 g); $n_{D}^{20}$ = 1.3834; IR (KBr, cm^−1^): 1786 (s), 1738 (s), 1456 (m), 1184 (s), 701 (m) cm^−1^; ^1^H-NMR (300 MHz, CDCl_3_) δ: 1.07 (t, *J* = 7.5 Hz, 3H, CH_3_CH_2_C(O)), 1.28 (d, *J* = 6.6 Hz, 3H, CH_3_-7), 2.17, 2.25 (two dq, *J* = 16.8, 7.5, 2H, CH_3_CH_2_C(O)), 2.65 (dd, *J* = 18.0, 8.1 Hz, 1H, one of CH_2_-3), 3.05 (dd, *J* = 18.0, 9.6 Hz, 1H, one of CH_2_-3), 3.58 (ddd, *J* = 9.6, 8.1, 6.6 Hz, 1H, H-4), 4.54 (dd, *J* = 6.6, 4.5 Hz, 1H, H-5), 5.15 (qd, *J* = 6.6, 4.5 Hz, 1H, H-6), 7.21–7.40 (m, 5H, H-2′, H-3′, H-4′, H-5′, H-6′); ^13^C-NMR (75 MHz, CDCl_3_) δ: 8.92 (CH_3_CH_2_C(O)), 15.80 (C-7), 27.62 (CH_3_CH_2_C(O)), 37.69 (C-3), 42.70 (C-4), 70.36 (C-6), 86.98 (C-5), 126.89 (C-2′, C-6′), 127.78 (C-4′), 129.33 (C-3′, C-5′), 140.65 (C-1′), 173.47 (CH_3_CH_2_C(O)), 175.31 (C-2); HRMS (ESI): *m*/*z* calcd. for C_15_H_18_O_4_ [M + H]^+^: 263.1283, found 263.1280.

*4-(4′-Methylphenyl)-5-(1-propionyloxyethyl)dihydrofuran-2-one* **6b**. Yield 82% (0.68 g), $n_{D}^{20}$ = 1.5542, IR (KBr, cm^−1^): 1786 (s), 1736 (s), 1457 (m), 1182 (s), 814 (m) cm^−1^; ^1^H-NMR (300 MHz, CDCl_3_) δ: 1.07 (t, *J* = 7.5 Hz, 3H, CH_3_CH_2_C(O)), 1.26 (d, *J* = 6.6 Hz, 3H, CH_3_-7), 2.18, 2.25 (two dq, *J* = 15.9, 7.5, 2H, CH_3_CH_2_C(O)), 2.33 (s, 3H, CH_3_-11), 2.63 (dd, *J* = 18.0, 7.8 Hz, 1H, one of CH_2_-3), 3.02 (dd, *J* = 18.0, 9.3 Hz, 1H, one of CH_2_-3), 3.54 (ddd, *J* = 9.3, 7.8, 6.6 Hz, 1H, H-4), 4.51 (dd, *J* = 6.6, 4.2 Hz, 1H, H-5), 5.13 (qd, *J* = 6.6, 4.2 Hz, 1H, H-6), 7.09–7.12 (m, 2H, H-2′, H-6′), 7.16–7.18 (m, 2H, H-3′, H-5′); ^13^C-NMR (75 MHz, CDCl_3_) δ: 8.93 (CH_3_CH_2_C(O)), 15.66 (C-7), 20.05 (C-11), 27.63 (CH_3_CH_2_C(O)), 37.71 (C-3), 42.33 (C-4), 70.30 (C-6), 87.12 (C-5), 126.77 (C-2′, C-6′), 129.94 (C-3′, C-5′), 137.52 (C-1′), 137.59 (C-4′), 173.47 (CH_3_CH_2_C(O)), 175.45 (C-2); HRMS (ESI): *m*/*z* calcd. for C_16_H_20_O_4_ [M + H]^+^: 277.1440, found 277.1447.

*4-(4′-Methoxyphenyl)-5-(1-propionyloxyethyl)dihydrofuran-2-one* **6c**. Yield 86% (0.75 g), $n_{D}^{20}$ = 1.5925, IR (KBr, cm^−1^): 1784 (s), 1736 (s), 1516 (s), 1253 (m), 1181 (s), 832 (m) cm^−1^; ^1^H-NMR (300 MHz, CDCl_3_) δ: 1.08 (t, *J* = 7.5 Hz, 3H, CH_3_CH_2_C(O)), 1.26 (d, *J* = 6.6 Hz, 3H, CH_3_-7), 2.19, 2.26 (two dq, *J* = 16.5, 7.5, 2H, CH_3_CH_2_C(O)), (2.61 (dd, *J* = 18.0, 8.1 Hz, 1H, one of CH_2_-3), 3.01 (dd, *J* = 18.0, 9.3 Hz, 1H, one of CH_2_-3), 3.53 (ddd, *J* = 9.3, 8.1, 6.6 Hz, 1H, H-4), 4.04 (s, 3H, CH_3_-11), 4.49 (dd, *J* = 6.6, 4.2 Hz, 1H, H-5), 5.13 (qd, *J* = 6.6, 4.2 Hz, 1H, H-6), 6.86–6.91 (m, 2H, H-3′, H-5′), 7.11–7.17 (m, 2H, H-2′, H-6′); ^13^C-NMR (75 MHz, CDCl_3_) δ: 8.94 (CH_3_CH_2_C(O)), 15.66 (C-7), 27.64 (CH_3_CH_2_C(O)), 3.78 (C-3), 41.9 (C-4), 55.38 (C-11), 70.25 (C-6), 87.16 (C-5), 114.64 (C-3′ and C-5′), 127.95 (C-2′ and C-6′), 132.45 (C-1′), 159.06 (C-4′), 173.49 (CH_3_CH_2_C(O)), 175.39 (C-2); HRMS (ESI): *m*/*z* calcd. for C_16_H_20_O_5_ [M + H]^+^: 293.1389, found 293.1386.

*4-(Benzo[d][1′,3′]dioxol-5′-yl)-5-(1-propionyloxyethyl)dihydrofuran-2-one* **6d**. Yield 88% (0.81 g), $n_{D}^{20}$ = 1.4941, IR (KBr, cm^−1^): 1782 (s), 1735 (s), 1490 (m), 1251 (s), 1188 (s), 1037 (s), 932 (m), 736 (m) cm^−1^; ^1^H-NMR (300 MHz, CDCl_3_) δ: 1,09 (t, *J* = 7.5 Hz, 3H, CH_3_CH_2_C(O)), 1.26 (d, *J* = 6.3 Hz, 3H, CH_3_-7), 2.21, 2.28 (two dq, *J* = 18.0, 7.8 Hz, 2H, CH_3_CH_2_C(O)), 2.59 (dd, *J* = 18.0, 7.8 Hz, 1H, one of CH_2_-3), 3.00 (dd, *J* = 18.0, 9.6 Hz, 1H, one of CH_2_-3), 3.50 (ddd, *J* = 9.6, 7.8, 6.6 Hz, 1H, H-4), 4.47 (dd, *J* = 6.6, 4.2 Hz, 1H, H-5), 5.13 (qd, *J* = 6.3, 4.2 Hz, H-6), 5.97 (s, 2H, CH_2_-2′) 6.67 (dd, *J* = 7.8, 1.5 Hz, 1H, H-6′), 6.70 (d, *J* = 1.5 Hz, 1H, H-4′), 6.78 (d, *J* = 7.8 Hz, 1H, H-7′); ^13^C-NMR (75 MHz, CDCl_3_) δ: 8.95 (CH_3_CH_2_C(O)), 15.65 (C-7), 27.66 (CH_3_CH_2_C(O)), 37.70 (C-3), 42.39 (C-4), 70.22 (C-6), 87.07 (C-5), 101.37 (C-2′), 106.96 (C-4′), 108.79 (C-7′), 120.22 (C-6′), 134.32 (C-5′), 147.13 (C-3′a), 148.46 (C-7′a), 173.48 (CH_3_CH_2_C(O)), 175.20 (C-2); HRMS (ESI): *m*/*z* calcd. for C_16_H_18_O_6_ [M + H]^+^: 307.1182, found 307.1188.

### 3.4. Enzymatic Resolution of Racemic Allyl Alcohol ***1c***

To the solution of racemic alcohol **1c** (5 g, 28 mmol) in DIPE (50 mL), 1 mL of vinyl propionate and lipase B from *C. antartica* (2 g) were added. The reaction was carried out in the 250 mL round-bottom flask on a magnetic stirrer at room temperature. The reaction was monitored by chiral GC after treating the samples with acetyl chloride to derivatize the unreacted alcohol into corresponding acetate **2**. Racemic acetate **2** and propionate **3** were used as the standards. After 4 h of reaction, the enzyme was filtered off and the organic solvent was evaporated in vacuo. Products of enzymatic tranesterification were separated by column chromatography (hexane/acetone, 15:1) and analyzed by chiral GC.

*(−)-(2S,3E)-4-(4′-Methoxyphenyl)but-3-en-2-ol (−)-***1c**. Yield (45%, 2.25 g); colourless crystals, *R*_f_ = 0.25 (hexane/acetone 4:1, *v*/*v*), m.p. 76–80 °C; *ee* = 78% (determined after derivatization into acetate); [α]${}_{D}^{20}$ = −28.5 (*c* 1.8, CH_2_Cl_2_) (lit. [40] [α]${}_{D}^{25}$ = −37.4 (*c* 0.84, CHCl_3_), *ee* = 99%), spectroscopic data identical to those reported for *rac-***1c** [31].

*(+)-(2R,3E)-4-(4′-Methoxyphenyl)but-3-en-2-yl propionate (+)-***3**. Yield 43% (2.82 g); pale-brown liquid, *R*_f_ = 0.66 (hexane/acetone 4:1, *v*/*v*), $n_{D}^{20}$ = 1.5263; *ee* = 82%, [α]${}_{D}^{20}$ = +113.5 (*c* 1.6, CH_2_Cl_2_); physical and spectroscopic data identical to those reported herein for *rac-***3**.

*Hydrolysis of (+)-(2R,3E)-4-(4′-methoxyphenyl)but-3-en-2-yl propionate*. Ester (+)-**3** (11 mmol) was hydrolyzed under reflux in 5% ethanolic solution of NaOH (40 mL). When the reaction was finished (3 h, TLC), ethanol was evaporated in vacuo and the residue was diluted with water. Alcohol (+)-**1c** was extracted with CH_2_Cl_2_ (3 × 30 mL) and the organic fractions were pooled, washed with brine until neutral and dried over anhydrous MgSO_4_. After evaporation of solvent in vacuo, pure alcohol (+)-**1c** was obtained.

(+)-(2*R*,3*E*)-4-(4′-Methoxyphenyl)but-3-en-2-ol (+)-**1c**. Yield 98% (1.61 g); colourless crystals, *ee* = 82%; [α]${}_{D}^{20}$ = +25.0 (*c* 1.6, CH_2_Cl_2_), (lit. [45] [α]${}_{D}^{23}$ = +30.68 (*c* 1.6, CHCl_3_), spectroscopic data identical to those reported for *rac*-**1c** [31].

### 3.5. Johnson–Claisen Rearrangement of Enantiomerically Enriched Allyl Alcohols (−)-***1c*** and (+)-***1c***

Mixture of enantiomerically enriched alcohol (−)-**1c** or (+)-**1c** (0.012 mol), triethyl orthoacetate (25 mL) and a drop of propionic acid was heated at 138–140 °C under reflux for 24 h with simultaneous removal of ethanol by distillation. The crude product was purified by column chromatography (hexane/acetone, 10:1, *v*/*v*).

*(+)-(3S,4E)-3-(4′-Methoxyphenyl)hex-4-enoic acid ethyl ester* (+)-**3c.** Obtained from alcohol (−)-(*S*)-**1c**. Yield 2.5 g (83%), *ee* = 78%, [α]${}_{D}^{20}$ = +8.8 (*c* 3.6, CH_2_Cl_2_), Physical and spectroscopic data are consistent with those of *rac*-**3c** [31].

*(−)-(3R,4E)-3-(4′-Methoxyphenyl)hex-4-enoic acid ethyl ester* (−)-**3c**. Obtained from alcohol (+)-(*R*)-**1c**. Yield 2.6 g (82%), *ee* = 82%, [α]${}_{D}^{20}$ = −9.4 (*c* 1.8, CH_2_Cl_2_); Physical and spectroscopic data are consistent with those of *rac*-**3c** [31].

### 3.6. Lactonization of Enantiomerically Enriched γ,δ-Unsaturated Esters (S)-***4a***–***d*** and (R)-***4a***–***d***

Enantiomerically enriched γ,δ-unsaturated ester (*S*)-**4a**–**d** or (*R)*-**4a**–**d** (0.007 mol) was dissolved in 50 mL of CHCl_3_ and *m*-CPBA (0.008 mol) and a drop of trifluoroacetic acid were added. The reaction mixture was stirred on a magnetic stirrer for 24 h. Then, the crude mixture was diluted with CHCl_3_ (100 mL), and successively washed with NaHSO_3_, NaHCO_3_ and brine. The combined organic layers were dried over anhydrous MgSO_4_ and filtered. The organic solvent was evaporated under vacuo and after column chromatography (silica gel, hexane/isopropanol/acetone/ethyl acetate/methylene chloride/diethyl ether (100:10:0.1:0.1:0.1:0.1, *v*/*v*/*v*/*v*/*v*/*v*) unseparable mixture of γ-hydroxy-δ-lactones and pure δ-hydroxy-γ-lactones **5a**–**d** were isolated. Their spectroscopic data were consistent those reported for their racemic form [33].

*(–)-trans-(4R,5S,6R)-5-(1-Hydroxyethyl)-4-phenyldihydrofuran-2-one* (−)-**5a**. Obtained from ester (+)-(4*E*,3*S*)-**4a**. Yield 0.12 g (8%), [α]${}_{D}^{20}$ = −19.0 (*c* = 3.25, CH_2_Cl_2_), *ee* = 90%.

*(+)-trans-(4S,5R,6S)-5-(1-Hydroxyethyl)-4-phenyldihydrofuran-2-one* (+)-**5a**. Obtained from ester (−)-(4*E*,3*R*)-**4a**. Yield 0.12 g (8%), [α]${}_{D}^{20}$ = +20.6 (*c* = 2.45, CH_2_Cl_2_), *ee* = 97%.

*(–)-trans-(4R,5S,6R)-5-(1-Hydroxyethyl)-4-(4′-methylphenyl)dihydrofuran-2-one* (−)-**5b**. Obtained from ester (+)-(4*E*,3*S*)-**4b**. Yield 0.12 g (8%), [α]${}_{D}^{20}$ = −16.5 (*c* = 5.0, CH_2_Cl_2_), *ee* = 83%.

*(+)-trans-(4S,5R,6S)-5-(1-Hydroxyethyl)-4-(4′-methylphenyl)dihydrofuran-2-one* (+)-**5b**. Obtained from ester (−)-(4*E*,3*R*)-**4b**. Yield 0.11 g (7%), [α]${}_{D}^{20}$ = + 17.5 (*c* = 2.14, CH_2_Cl_2_), *ee* = 87%.

*(–)-trans-(4R,5S,6R)-5-(1-Hydroxyethyl)-4-(4′-methoxyphenyl)dihydrofuran-2-one* (−)-**5c**. Obtained from ester (+)-(4*E*,3*S*)-**4c**. Yield 0.12 g (7%), [α]${}_{D}^{20}$ = −8.3 (*c* = 3.53, CH_2_Cl_2_), *ee* = 78%.

*(+)-trans-(4S,5R,6S)-5-(1-Hydroxyethyl)-4-(4′-methoxyphenyl)dihydrofuran-2-one* (+)-**5c.** Obtained from ester (−)-(4*E*,3*R*)-**4c**. Yield 0.12 g (8%), [α]${}_{D}^{20}$ = +8.9 (*c* = 2.0, CH_2_Cl_2_), *ee* = 82%.

*(–)-trans-(4R,5S,6R)-4-(Benzo[d][1′,3′]dioxol-5′-yl)-5-(1-hydroxyethyl)-dihydrofuran-2-one* (−)-**5d.** Obtained from ester (−)-(4*E*,3*R*)-**4d**. Yield 0.13 g (7.5%), [α]${}_{D}^{20}$ = −6.3 (*c* = 4.40, CH_2_Cl_2_), *ee* = 73%.

*(+)-trans-(4S,5R,6S)-4-(Benzo[d][1′,3′]dioxol-5′-yl)-5-(1-hydroxyethyl)-dihydrofuran-2-one* (+)-**5d**. Obtained from ester (+)-(4*E*,3*S*)-**4d**. Yield 0.14 g (8%), [α]${}_{D}^{20}$ = +6.7 (*c* = 2.58, CH_2_Cl_2_), *ee* = 82%.

### 3.7. Enzymatic Resolution of Hydroxylactones ***5a***–***d***

#### 3.7.1. Screening Procedure

To a solution of hydroxylactone **5a** in 10 mL of DIPE 5 mg of lipase and 0.1 mL of vinyl propionate was added. The reaction mixture was stirred in a magnetic stirrer in 20 mL-vial at room temperature. At several time intervals, the samples (0.5 mL) were withdrawn from reaction mixture and filtered through Celite 560. The organic solvent was evaporated under vacuo, and the residue was dissolved in acetone (0.2 mL) and analyzed by CGC. The results are shown in Table 2.

#### 3.7.2. Preparative Transesterification

To a solution of β-aryl-δ-hydroxy-γ-lactone **5a**–**d** (0.97 mmol) in 50 mL of DIPE, 100 mg of lipase B from *Candida antarctica* (CAL-B) and 1 mL of vinyl propionate were added and the mixture was stirred at room temperature. The reaction was monitored by chiral GC and racemic hydroxylactones **5a**–**d** and propionates **6a**–**d** were used as the standards. After 6 h the enzyme was filtered off and organic solvent was evaporated in vacuo. Slowly reacting hydroxylactones **5a**–**d** and propionates **6a**–**d** were separated by column chromatography (hexane/acetone, 20:1, *v*/*v*). Their physical and spectroscopic data were in accordance with those of racemic forms of lactones [33] and propionates reported herein (Section 3.3).

*(+)-trans-(4S,5R,6S)-5-(1-Hydroxyethyl)-4-phenyldihydrofuran-2-one* (+)-**5a**. Yield 0.08 g (38%), *R*_f_ = 0.21 (hexane/acetone 4:1, *v*/*v*), [α]${}_{D}^{20}$ = +21.0 (*c* = 0.55, CH_2_Cl_2_), *ee* = 99%.

*(+)-trans-(4R,5S,6R)-4-Phenyl-5-(1-propionyloxyethyl)-dihydrofuran-2-one* (+)-**6a**. Yield 0.11 g (44%), *R*_f_ = 0.71 (hexane/acetone 4:1, *v*/*v*), [α]${}_{D}^{20}$ = + 33.7 (*c* = 0.60, CH_2_Cl_2_), *ee* = 92%.

*(+)-trans-(4S,5R,6S)-5-(1-Hydroxyethyl)-4-(4′-methylphenyl)dihydrofuran-2-one* (+)-**5b**. Yield 0.09 g (41%), *R*_f_ = 0.22 (hexane/acetone 4:1, *v*/*v*), [α]${}_{D}^{20}$ = +16.9 (*c* = 0.40, CH_2_Cl_2_), *ee* = 99%.

*(+)-trans-(4R,5S,6R)-4-(4′-Methylphenyl)-5-(1-propionyloxyethyl)-dihydrofuran-2-one* (+)-**6b**. Yield 0.12 g (44%), *R*_f_ = 0.68 (hexane/acetone 4:1, *v*/*v*), [α]${}_{D}^{20}$ = +30.1 (*c* = 0.65, CH_2_Cl_2_), *ee* = 91%.

*(+)-trans-(4S,5R,6S)-5-(1-Hydroxyethyl)-4-(4′-methoxyphenyl)dihydrofuran-2-one* (+)-**5c**. Yield 0.103 g (45%), *R*_f_ = 0.27 (hexane/acetone 4:1, *v*/*v*), [α]${}_{D}^{20}$ = +9.5 (*c* = 0.40, CH_2_Cl_2_), *ee* = 98%.

*(+)-trans-(4R,5S,6R)-4-(4′-Methoxyphenyl)-5-(1-propionyloxyethyl)-dihydrofuran-2-one* (+)-**6c**. Yield 0.10 g (36%), *R*_f_ = 0.67 (hexane/acetone 4:1, *v*/*v*), [α]${}_{D}^{20}$ = +14.4 (*c* = 2.8, CH_2_Cl_2_), *ee* = 98%.

*(+)-trans-(4S,5R,6S)-4-(Benzo[d][1′,3′]dioxol-5′-yl)-5-(1-hydroxyethyl)-dihydrofuran-2-one* (+)-**5d**. Yield 0.08 g (34%), *R*_f_ = 0.23 (hexane/acetone 4:1, *v*/*v*), [α]${}_{D}^{20}$ = +6.8 (*c* = 0.40, CH_2_Cl_2_), *ee* = 89%.

*(+)-trans-(4R,5S,6R)-4-(Benzo[d][1′,3′]dioxol-5′-yl)-5-(1-propionyloxyethyl)-dihydrofuran-2-one* (+)-**6d.** Yield 0.10 g (35%), *R*_f_ = 0.70 (hexane/acetone 4:1, *v*/*v*), [α]${}_{D}^{20}$ = +15.6 (*c* = 0.80, CH_2_Cl_2_), *ee* = 84%.

## 4. Conclusions

In conclusion, we have presented two alternative methods for the production of enantiomerically enriched *trans*-β-aryl-δ-hydroxy-γ-lactones **5a**–**d** with the defined absolute configurations of their stereogenic centers, which were not reported previously in optically active forms. Chemoenzymatic synthesis gives direct access to both enantiomers of desired lactones (*ee* = 73%–97%), and kinetic resolution of their racemic mixtures by CAL-B delivers (+)-enantiomers **5a**–**d** and (+)-propionates **6a**–**d,** which can be further hydrolyzed to (−)-enantiomers of desired hydroxylactones. Most of the products of enzymatic reaction were obtained with excellent enantiomeric excesses (*ee* = 91%–99%). 1,3-Benzodioxol ring as the steric hindrance lowered enantioselectivity of resolution of lactone **5d**. The methods presented above are convenient routes to obtain both enantiomers of optically active *trans*-β-aryl-δ-hydroxy-γ-lactones **5a**–**d,** which will let us study the dependence between their spatial structure and biological activity.

## Figures and Tables

**Table 1:** Results of enzymatic transesterification of *rac*-δ-hydroxy-γ-lactone **5a** with vinyl propionate.

Enzyme	Time (h)	Conversion ^a^ (mol %)	*ee* of (+)-Hydroxylactone 5a (%)	*ee* of (+)-Propionyloxylactone 6a (%)	*E* ^b^
CAL-B	1	49	96	99	>200
	2	51	99	96	>200
	4	51	99	96	>200
	6	51	99	96	>200
CCL	2	17	20	97	80
	4	34	42	80	13
	6	44	55	71	10
Lipozyme TL IM	2	11	12	99	>200
	4	43	68	90	38
	6	53	91	80	28
Amano Lipase PS	2	11	12	99	>200
	4	25	34	99	>200
	6	40	65	99	>200

^a^ Conversion calculated according to equation: c = (*ee_s_*/*ee_s_* + *ee_p_)* × 100; ^b^ The enantiomeric ratio calculated according to equation: *E* = ln[(1 − *ee*_s_)/(1 + (*ee*_s_/*ee*_p_)]/ln[(1 + *ee*_s_)/(1 + (*ee*_s_/*ee*_p_)]; *ee*_s_ = enantiomeric excess of unreacted hydroxylactone **5a**, *ee*_p_ = enantiomeric excess of propionate **6a**.

**Table 2:** Results of CAL-B-catalyzed preparative transesterification of *rac*-δ-hydroxy-γ-lactones **5b**–**d** with vinyl propionate after 6 h of process.

Substrate	Conversion ^a^ (mol %)	*ee* of (+)-Hydroxylactone (%)	*ee* of (+)-Propionyloxylactone (%)	*E* ^b^
**5b**	52	99 (**5b**)	91 (**6b**)	111
**5c**	50	98 (**5c**)	98 (**6c**)	>200
**5d**	51	89 (**5d**)	84 (**6d**)	34

^a^ Conversion calculated according to equation: c = (*ee_s_*/*ee_s_* + *ee_p_*) × 100; ^b^ The enantiomeric ratio calculated according to equation: *E* = ln[(1 − *ee*_s_)/(1 + (*ee*_s_/*ee*_p_)]/ln[(1 + *ee*_s_)/(1 + (*ee*_s_/*ee*_p_)]; *ee*_s_ = enantiomeric excess of unreacted hydroxylactones **5b**–**d**, *ee*_p_ = enantiomeric excess of propionates **6b**–**d**.

## Supplementary Materials

Supplementary materials can be accessed at: http://www.mdpi.com/1420-3049/21/11/1552/s1.
