# Supplementary material for: Chemoenzymatic Synthesis of trans-β-Aryl-δ-hydroxy-γ-lactones and Enzymatic Kinetic Resolution of Their Racemic Mixtures
Source: Molecules. 2016 Nov 23;21(11):1552. doi: 10.3390/molecules21111552 (PMC6272857; doi:10.3390/molecules21111552)
Supplement: Supplementary file 1 [file molecules-21-01552-s001.pdf]

Supplementary Materials: Chemoenzymatic Synthesis of *trans*-β-Aryl-δ-hydroxy-γ-lactones and Enzymatic Kinetic Resolution of Their Racemic Mixtures

Andrzej Skrobiszewski, Witold Gładkowski, Gabriela Maciejewska and Czesław Wawrzeńczyk

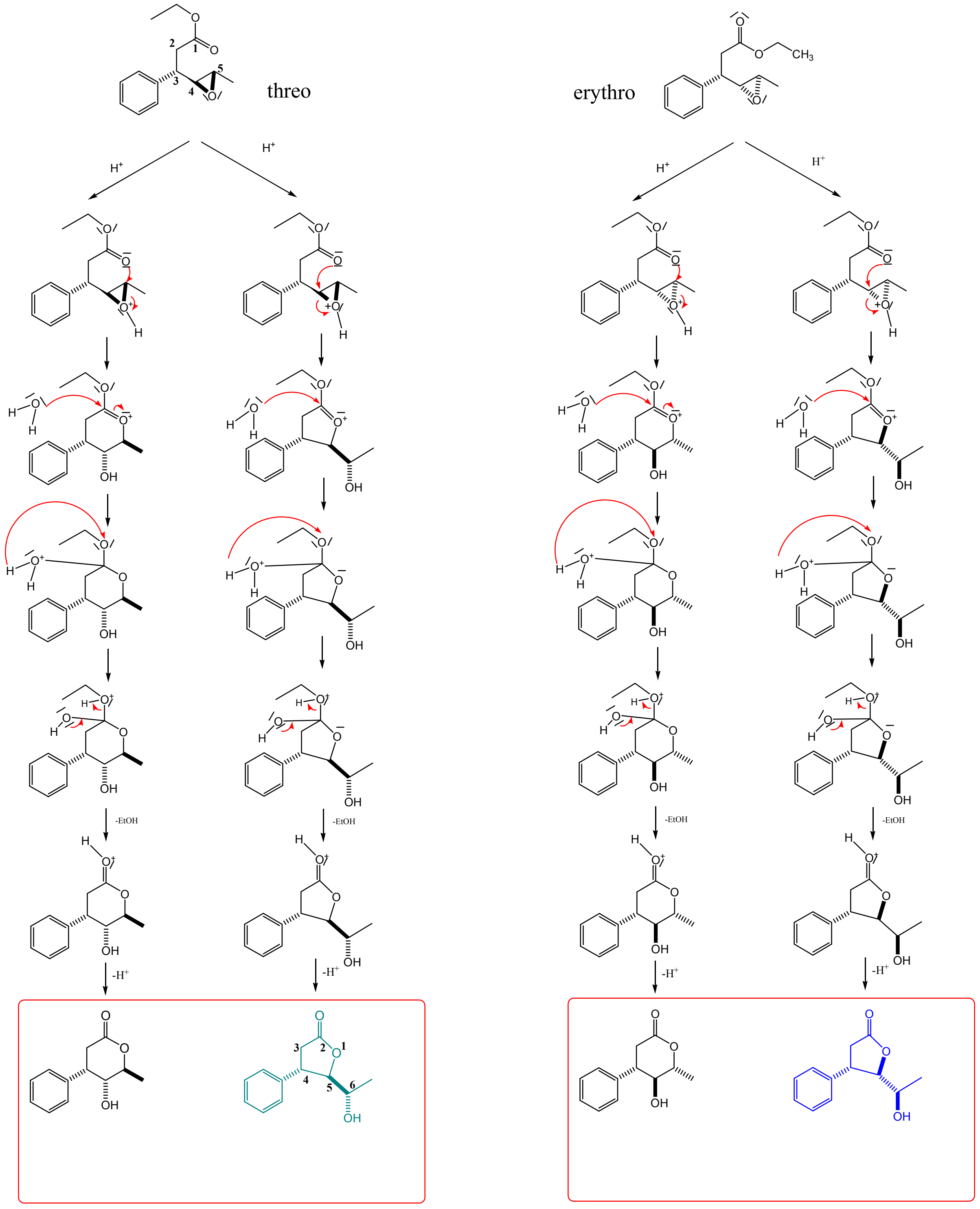

Figure S1. Formation of possible product of lactonization of *threo* and *erythro* isomers of epoxyesters.
